# Relationship Between Neighborhood Food Environment and Diet Variety in Japanese Rural Community-dwelling Elderly: A Cross-sectional Study

**DOI:** 10.2188/jea.JE20200415

**Published:** 2022-06-05

**Authors:** Tatsunosuke Gomi, Jun Kitayuguchi, Kenta Okuyama, Masamitsu Kamada, Shigeru Inoue, Hiroharu Kamioka, Yoshiteru Mutoh

**Affiliations:** 1 Physical Education and Medicine Research Center UNNAN, Unnan, Shimane, Japan; 2 Department of Environmental Symbiotic Studies, Tokyo University of Agriculture, Tokyo, Japan; 3 Center for Primary Health Care Research, Lund University, Malmö, Sweden; 4 Department of Health and Social Behavior, School of Public Health, Graduate School of Medicine, The University of Tokyo, Tokyo, Japan; 5 Department of Preventive Medicine and Public Health, Tokyo Medical University, Tokyo, Japan; 6 The Research Institute of Health Rehabilitation of Tokyo, Tokyo, Japan

**Keywords:** diet variety, neighborhood food environment, public health, geographic information systems

## Abstract

**Background:**

Food access is an important aspect of health promotion for the elderly. The aim of this study was to investigate the relationship between distance to the nearest food store and diet variety in rural community-dwelling elderly Japanese.

**Methods:**

This cross-sectional study analyzed data from 1,103 elderly participants surveyed by mail in rural areas of Japan. Diversity of food intake was assessed using the diet variety score (DVS). Street network distance from home to food store was calculated and categorized by quartile using a geographic information system and analyzed in relation to diet using multivariable regression with the primary outcome as low DVS. Sub-analysis of the association with DVS was conducted for each food store category (convenience store, supermarket, and small food store). The association between intake frequency of each food group and distance was also analyzed.

**Results:**

Participants in the fourth quartile of distance to food store had significantly higher prevalence ratio (1.15; 95% CI, 1.01–1.32) for low DVS than those in the first quartile. There was a significant tendency between greater distance to food store and lower DVS (*P* for trend = 0.033). Supermarkets and convenience stores, in particular, showed significant associations. Greater distance was significantly associated with lower frequency of meat and fruit intake.

**Conclusion:**

There was significant association between distance to nearest food store and diet variety in rural Japanese elderly. These findings suggest the importance of interventions for areas at high risk of low diet variety, such as places far away from food stores.

## INTRODUCTION

Frailty among the elderly is a major problem worldwide.^1^ Japan, the most rapidly aging society in the world, China, Canada, and many European countries will have aging population rates greater than 30% by 2050.^2^

One cause of frailty is lack of nutrients and calories. Nutritional deficiencies specific to the elderly are due to factors such as anorexia of aging,^3^ cognitive impairment,^4^ mental health symptoms,^4^^,^^5^ and oral dysphagia.^6^ Additionally, changing lifestyles in Japan and decreased numbers of retail food stores have made it more difficult for people to access foods.^7^ Several studies have been conducted on food environment and healthy eating^8^: obesity^9^ and type 2 diabetes^10^ were associated with food accessibility among adults in the United States, which led to interventions such as increasing the number of grocery stores.^11^ However, there is little research on the relationship between diets of elderly people and food environments. Low diet variability was reportedly related to frailty,^12^ lean mass,^13^ and physical function^14^ in a study of Japan’s elderly and efforts are focused on expanding diet variety to maintain and improve health. The previous study reported that distance to the nearest supermarket was not associated with diet variety,^12^ whereas Harada et al showed that it was significantly associated with lower diet variety.^15^ The contradiction between these two studies may be that only one type of food store, supermarket, was examined and not the whole food environment. No study has examined the relationship between diet variety and neighborhood food environment, including small retail food and convenience stores; this has limited efforts to conduct dietary interventions with an environmental-level approach towards improving diets in the elderly.

In this study, we hypothesized that poor accessibility including all type of food store associate with lower diet variety among Japanese elderly. We aimed to investigate the relationship between distance to the nearest food store and diet variety.

## METHODS

### Data collection

We evaluated cross-sectional observations from an ongoing community-level intervention study designed to improve levels of physical activity (UMIN000024682). The intervention study began in 2016 with baseline surveys conducted on 3,310 residents aged 63 to 79 years who were randomly selected from the registry of Unnan City (population 37,416, area 553.4 km^2^), a rural mountainous region in Shimane, Japan.

In November 2018, two years after the baseline survey, we mailed the second time survey questionnaire only to subjects who had consented and responded to the baseline survey. Postcard reminders were sent to non-responders to increase the response rate. Written informed consent was obtained from each subject who agreed to participate, and an opt-out option for the analysis was added to the research institution’s homepage because our analysis protocol was not included in the initial intervention study. We set exclusion criteria of needing someone’s assistance to go outside. The present study was approved by the research ethics committee of the Physical Education and Medicine Research Center UNNAN (R1-7-2-2).

### Assessment of dietary variety

Dietary variety was assessed by a dietary variety score (DVS) developed by Kumagai et al.^16^ The DVS is a food-based composite score determined by calculating consumption frequencies for 10 food groups that constitute a large part of Japanese daily main and side dishes: meat, fish/shellfish, eggs, milk, soybean products, green/yellow vegetables, potatoes, fruit, seaweed, and fats/oils. Respondents were asked about consumption frequencies during one week for each of the 10 food items. A score of 1 was assigned for “eat almost every day”, and a score of 0 was assigned for “not eat every day”. Total scores ranged from 0 to 10, and higher scores indicated greater diet variety. A previous study reported a significant trend between low DVS and low intakes of legumes, green-yellow vegetables, fruits, and eggs, and significant trends between lower intakes of energy, protein and fat and lower DVS.^17^ In addition, the group with DVS ≤2 had a significantly higher odds ratio with frailty when referenced to groups with DVS ≥5.^17^ It was also reported that a group with DVS ≤2 was significantly related to lower lean mass and grip strength in Japanese elderly.^14^ We categorized DVS ≤2 as “low”. Intake frequency for each food group was categorized as “low” if it was once every two days or less.

### Neighborhood food environment

Geographic information system (GIS; ArcGIS 10.5.1 software from ESRI Corporation, Redlands, CA, USA) was used to estimate road network distance between each participant’s actual residential location and nearest food store. Neighborhood food environments have been reported to have different effects on residents, depending on the type of food store.^18^ In order to consider which food store was more important to each older adult, interpretation of the results was performed using supermarkets, convenience stores, and all other small food stores in the sub-analyses. Location data for food stores, bus stops, and stations as of March 2018 were obtained from a geographical database (Zmap-AREA II Chugoku region, Zenrin Corporation, Fukuoka, Japan). Road network distance between a participant’s location and nearest food store was based on 2016 data (National Digital Road Map Database, Japan Digital Road Map Association, Tokyo, Japan). Distances to nearest food store were categorized into quartiles: Q1, <329 m; Q2, 329–841 m; Q3, 842–1,783 m; and Q4, 1,784–7,780 m. Respective distances to supermarkets, convenience stores, and small food stores were also categorized into quartiles. Road network distances from participant’s homes to nearest bus stops and stations were calculated using GIS. Food environment was measured subjectively via questionnaire; participants were asked, “Have you eaten any in-house garden vegetables in the past year?”, and were to answer either “often”, “sometimes”, “little”, or “never”. Answers were dichotomized as yes (often) or no (sometimes, little, or never). Participants were also asked, “Is usually getting your groceries easy?”, and were to answer either “very easy”, “not bad”, “a little hard”, or “very hard”. Answers were dichotomized as high (very easy) or low (not bad, a little hard, or very hard).

### Covariates

Covariates included sex, age, body mass index (BMI; computed as subjectively measured weight in kilograms divided by height in meters squared), current medical history (hypertension, hyperlipemia, diabetes, hyperuricemia, stroke, heart disease, vascular disease, kidney disease, hepatic disease, gastrointestinal disease, endocrine disease, osseous disease, cancer), medication use (never, using one to four, or using five or more), depression symptoms, chewing ability, smoking habit (no smoking or current smoker), physical activity levels, living alone, food service use, years of education, employment, and driving status. Age was categorized as 60–69 years (60s), or ≥70 years (70s or older). BMI was categorized using the 2020 Dietary Reference Intakes for Japanese ≥65 years of age.^19^ Number of medications used was categorized to account for polypharmacy.^20^ The presence of depressive symptoms was assessed using the Kessler Screening Scale for Psychological Distress (K6),^21^ which measures general psychological distress, including depression and anxiety. We used the Japanese version of the K6, which demonstrated screening performances essentially equivalent to those previously reported for the original English versions.^22^ In our study, severe depressive symptoms were indicated by a K6 score of ≥5. Self-perceived chewing ability was classified as “moderate” for participants who answered, “can chew anything” and “low” for participants who answered, “there is something cannot chew” or “do not chew much” or “eat blended food”. Smoking was categorized as “yes” for participants who currently smoked and “no” for participants who were former or never smokers. The Japanese version of the International Physical Activity Questionnaire-short version (IPAQ-SV) was used to evaluate and distribute physical activity levels into three categories defined by the IPAQ.^23^ Participants were asked, “who do you currently live with?”, and answers were categorized as “live alone” or “other”. Participants were asked whether they used a “meal delivery service”, or “home-delivered lunches”, or “food stuff delivery”, or “mobile sales”, or “not used”, and answers were categorized as “used at least one meal service” or “never”. Educational levels were divided into ≤12 years or >12 years. Participants were also asked, “do you have a driving license?”, and answers were categorized as “yes” or “no”. Employment was categorized as “yes” for participants who worked one day or more with income and “no” for other participants.

### Statistical analyses

Prevalence of all variables was described according to distance to nearest food store. The main analysis, prevalence ratio (PR) and 95% confidence interval (CI), was derived from multivariate adjusted Poisson regression for diet variety and distance to nearest food store. As sub-analyses, the same model was performed separately for distance to supermarket, convenience store, and small food store. Distances to bus stop and railway station, in-house garden vegetables, and perceived access to food were also analyzed. Odds ratio calculated using binomial regression analysis to estimate risk ratio is known to overestimate or underestimate if the incidence of cases is >10%.^24^ Therefore, we used PR calculated using multivariate Poisson regression analysis modified using sandwich estimation,^25^ which previous reports have shown does not cause overestimation and underestimation whenever incidence of outcomes is high.^26^^,^^27^ Analyses were performed with the unadjusted model (crude) and the model adjusted for sex, age, BMI, disease history, medication use, depressive symptoms, perceived chewing ability, smoking, physical activity levels, living alone, food service use, years of education, employment status, and driving license (adjusted model). These covariates were included because they are related to dietary habits^6^ and are treated as adjustment factors in epidemiological nutrition studies.^28^^,^^29^ Food service use and driving license also affect food access and were therefore included as covariates. Each parameter of these covariates is shown in eTable 1.

Multiple linear regression sensitivity analysis was performed by treating DVS and distance to nearest food store as continuous variables with the same covariates as above. We conducted stratified analyses by sex and driving status with a Poisson regression model.

While DVS itself is important, it is also important to know which food group elderly people have difficulties in accessing; therefore, we performed multivariate Poisson regression analysis of each food group that had cases with a “low” intake frequency, using covariates identical to those in the main analysis.

For all Poisson regression, multicollinearity was examined with all variables, but none showed enough correlation to cause multicollinearity (variance inflation factor: VIF <2.3, data not shown). In addition, we calculated the ratio of the Pearson chi-square statistic to its degrees of freedom (value/df) to determine the presence or absence of overdispersion for all Poisson regression models. The value/df of all models were less than 1.0 (the highest value/df was 0.562, data not shown). We therefore confirmed that there was no overdispersion, and the goodness of fit was acceptable in each model.^30^ Because participants with missing values were excluded from analyses, there was no missing value in the statistical analyses. All statistical analyses were performed with SPSS Statistics 25.0 (IBM Corporation, Armonk, NY, USA). *P*-values less than 0.05 were considered statistically significant.

Research procedures, analyses, and explanations were reported according to the Strengthening the Reporting of Observational Studies in Epidemiology (STROBE) statement.^31^

## RESULTS

**Figure 1.**
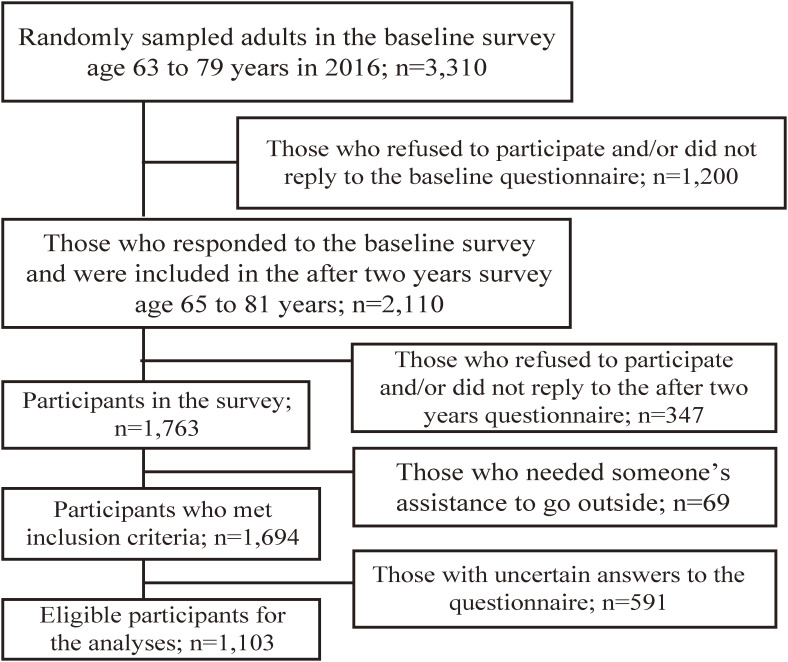
Study flow

From the 3,310 adults who responded to the original 2016 community-level intervention study, 2,110 men and women who were age 65 to 81 years in 2018 were invited to participate in the present study. Of those invited, 1,763 (53.3%) consented to participate (Figure 1); however, 69 respondents were subsequently excluded because they met the exclusion criteria. Another 591 respondents with uncertain or missing answers to the questions were excluded. Consequently, data from 1,103 participants were analyzed.

Participant characteristics are described in Table 1. The proportion of low DVS was 59.4%, and median DVS was 3 (2–5 Q1 to Q3). Median distance to nearest food store for all participants was 840 m (329–1,782 m Q1 to Q3).

**Table 1.** Characteristics of participants according to distance to the nearest food store by quartiles

		Distance to the nearest food store by quartile			
	Total (*n* = 1,103)	Q1(<329 m) (*n* = 276)	Q2(<841 m) (*n* = 276)	Q3(<1,783 m) (*n* = 276)	Q4(<7,780 m) (*n* = 275)
Dietary variety score	3 (2–5)	3 (2–5)	3 (2–5)	3 (2–5)	3 (2–4)
Low (≤2)	655 (59.4)	155 (56.2)	153 (55.4)	165 (59.8)	182 (66.2)
Sex, female	576 (52.2)	150 (54.3)	156 (56.5)	139 (50.4)	131 (47.6)
Age, years	70 (68–75)	71 (68–75)	70 (68–75)	70 (68–74)	70 (67–75)
60s	454 (41.2)	104 (37.7)	109 (39.5)	122 (44.2)	119 (43.3)
70s or older	649 (58.8)	172 (62.3)	167 (60.5)	154 (55.8)	156 (56.7)
Body mass index, kg/m^2^	22.4 (20.7–24.3)	22.5 (20.7–24.2)	22.3 (20.5–24.2)	22.5 (20.6–24.4)	22.3 (20.7–24.5)
≤21.4	403 (36.5)	96 (34.8)	109 (39.5)	98 (35.5)	100 (36.4)
21.5–24.9	500 (45.3)	135 (48.9)	123 (44.6)	124 (44.9)	118 (42.9)
≥25.0	200 (18.1)	45 (16.3)	44 (15.9)	54 (19.6)	57 (20.7)
Disease history, yes	831 (75.3)	198 (71.7)	198 (71.7)	218 (79.0)	217 (78.9)
Medication use					
None	231 (20.9)	53 (19.2)	65 (23.6)	55 (19.9)	58 (21.1)
0–4	658 (59.7)	172 (62.3)	153 (55.4)	166 (60.1)	167 (60.7)
5 or over	214 (19.4)	51 (23.8)	58 (27.1)	55 (25.7)	50 (23.4)
Depressive symptoms, yes	174 (15.8)	41 (14.9)	38 (13.8)	44 (15.9)	51 (18.5)
Perceived chewing ability, low	405 (36.7)	93 (33.7)	87 (31.5)	108 (39.1)	117 (42.5)
Smoking, yes	98 (8.9)	22 (8.0)	23 (8.3)	32 (11.6)	21 (7.6)
Physical activity levels					
Low	809 (73.3)	220 (79.7)	203 (73.6)	206 (74.6)	180 (65.5)
Modelate	226 (20.5)	46 (16.7)	54 (19.6)	57 (20.7)	69 (25.1)
High	68 (6.2)	10 (3.6)	19 (6.9)	13 (4.7)	26 (9.5)
Living alone, yes	79 (7.2)	24 (8.7)	19 (6.9)	15 (5.4)	21 (7.6)
Food service use, yes	60 (5.4)	6 (2.2)	17 (6.2)	18 (6.5)	19 (6.9)
Years of education, years	12 (9–12)	12 (10–13)	12 (9–12)	12 (9–12)	11 (9–12)
More than 12 years	194 (17.6)	74 (26.8)	39 (14.1)	48 (17.4)	33 (12.0)
Employment, yes	504 (45.7)	123 (44.6)	116 (42.0)	126 (45.7)	139 (50.5)
Driving license, yes	971 (88.0)	230 (83.3)	241 (87.3)	247 (89.5)	253 (92.0)
Distance to the nearest food store, m	840 (329–1782)	197 (95–256)	555 (446–671)	1182 (996–1437)	3208 (2438–4077)
Distance to the nearest bus stop, m	270 (140–486)	155 (78–235)	303 (167–452)	320 (161–513)	476 (243–1561)
Distance to the nearest station, m	2546 (1236–6145)	1152 (430–2708)	1828 (933–3861)	2293 (1644–4465)	6244 (3675–10358)
In-house vegetable garden, yes	783 (71.0)	151 (54.7)	186 (67.4)	217 (78.6)	229 (83.3)
Perceived access to foods, low	65 (5.9)	13 (4.7)	10 (3.6)	13 (4.7)	29 (10.5)

Q, quartile.

Table shows median (quartile 1, quartile 3) or *n* (%) where appropriate.

In multivariate analysis by distance to nearest food store (Table 2), there was a significantly higher PR (1.15; 95% CI, 1.01–1.32) for low DVS only in Q4 (1,784 m–7,780 m). There was also a significant trend (*P* for trend = 0.033) between low DVS and distance to nearest food store. Results were similar for analyses in the crude model. The Q4 of the distances to nearest supermarket, convenience store, and small food store had a higher PR of low DVS (Table 2). There was a significant trend for high PR of low DVS to increase with greater distance to each type of store. In the adjusted model, Q4 of the distance to nearest supermarket had a significantly higher PR than Q1. Moreover, there was a significant tendency for risk of lower DVS with greater distance to supermarket and convenience store; small food stores were not significantly related to low DVS in the adjusted model. Greater distance to nearest bus stop was significantly related to low DVS in the crude model, but not in the adjusted model. Greater distance to nearest railway station was significantly related to low DVS in both the crude and adjusted models. No significant association was detected between having in-house garden vegetables or perceived access to foods and low DVS.

**Table 2.** Prevalence ratio (95% confidence interval) for the low diet variety score (DVS) for neighborhood food environment

	No. adults with DVS ≤2 (%)	Crude	Adjusted model^a^
*n* = 1,103		PR (95% CI)^b^	PR (95% CI)^b^
Distance to the nearest food store			
Q1 (<329 m)	56.2	1 (ref.)	1 (ref.)
Q2 (329–841 m)	55.4	0.99 (0.85–1.15)	1.01 (0.87–1.17)
Q3 (842–1,783 m)	59.8	1.07 (0.92–1.23)	1.04 (0.91–1.20)
Q4 (1,784–7,780 m)	66.2	1.18 (1.03–1.35)^*^	1.15 (1.01–1.32)^*^
*P* for trend		0.009	0.033
Distance to the nearest supermarket			
Q1 (<875 m)	54.7	1 (ref.)	1 (ref.)
Q2 (875–2,184 m)	57.2	1.05 (0.90–1.21)	1.02 (0.88–1.18)
Q3 (2,185–4,094 m)	58.7	1.07 (0.93–1.24)	1.06 (0.92–1.22)
Q4 (4,095–16,613 m)	66.9	1.22 (1.07–1.40)^**^	1.18 (1.03–1.35)^*^
*P* for trend		0.004	0.011
Distance to the nearest convenience store			
Q1 (<1,129 m)	56.2	1 (ref.)	1 (ref.)
Q2 (1,130–2,486 m)	55.1	0.98 (0.85–1.14)	0.98 (0.85–1.13)
Q3 (2,487–4,720 m)	61.2	1.09 (0.95–1.26)	1.06 (0.93–1.22)
Q4 (4,721–17,638 m)	65.1	1.16 (1.01–1.33)^*^	1.13 (0.99–1.29)
*P* for trend		0.013	0.036
Distance to the nearest small food store			
Q1 (<385 m)	55.1	1 (ref.)	1 (ref.)
Q2 (386–956 m)	57.6	1.05 (0.9–1.21)	1.05 (0.91–1.22)
Q3 (957–1,890 m)	59.4	1.08 (0.93–1.25)	1.06 (0.92–1.22)
Q4 (1,891–8,022 m)	65.5	1.19 (1.04–1.36)^*^	1.14 (1.00–1.31)
*P* for trend		0.013	0.063
Distance to the nearest bus stop/km		1.06 (1.01–1.12)^*^	1.05 (0.99–1.11)
Distance to the nearest station/km		1.01 (1.00–1.02)^**^	1.01 (1.00–1.02)^*^
In-house vegetable garden			
No	63.8	1 (ref.)	1 (ref.)
Yes	57.6	0.9 (0.82–1.00)	0.93 (0.84–1.03)
Perceived access to foods			
High	58.8	1 (ref.)	1 (ref.)
Low	69.2	1.18 (0.99–1.40)	1.11 (0.95–1.31)

CI, confidence interval; DVS, Diet variety score; PR, prevalence ratio; Q, quartile.

^a^Adjusted for sex, age, body mass index, disease history, medication use, depressive symptoms, perceived chewing ability, smoking, physical activity levels, living alone, food service use, years of education, employment status and driving license. ^b^Prevalence ratio calculated by Poisson regression. ^*^P < 0.05, ^**^P < 0.01.

Sensitivity analyses detected a significant relationship between DVS and distance to all food stores (β = −0.068, *P* = 0.021), convenience store (β = −0.061, *P* = 0.036), and small food store (β = −0.063, *P* = 0.032), all treated as continuous valuables. There was no significant relationship between DVS and distance to supermarket (β = −0.057, *P* = 0.053).

Frequency of meat consumption was significantly low in Q2 and Q4 for distance to nearest food store (Table 3). There was a significant trend towards increased PR for low frequency of fruit intake with increased distance to nearest food store (*P* for trend <0.001). Additionally, low fruit intake had a significantly high PR in Q4 according to distance to food store (PR 1.30; 95% CI, 1.12–1.50).

**Table 3.** Prevalence ratio (95% confidence interval) for the low frequency (once every two days or less) of consumption of each food group to distance to the nearest food store

	No. adults with “low” (%)	Distance to the nearest food store by quartile				
		Q1 (<329 m)	Q2 (<841 m)	Q3 (<1,783 m)	Q4 (<7,780 m)	
		(*n* = 276)	(*n* = 276)	(*n* = 276)	(*n* = 275)	
*n* = 1,103			PR (95% CI)^a^	PR (95% CI)^a^	PR (95% CI)^a^	*P* for trend^a^
Fish/shellfish	71.2	1 (ref.)	1.01 (0.90–1.12)	1.05 (0.95–1.17)	1.03 (0.93–1.15)	0.427
Meat	81.2	1 (ref.)	1.10 (1.01–1.20)^*^	1.06 (0.97–1.15)	1.10 (1.02–1.20)^*^	0.058
Eggs	58.6	1 (ref.)	0.97 (0.85–1.11)	0.91 (0.79–1.05)	0.93 (0.81–1.07)	0.221
Milk	55.8	1 (ref.)	0.97 (0.84–1.12)	0.97 (0.84–1.12)	0.95 (0.82–1.10)	0.508
Soybean products	64.8	1 (ref.)	0.98 (0.86–1.11)	1.05 (0.93–1.18)	1.02 (0.90–1.16)	0.489
Green/yellow vegetables	44.9	1 (ref.)	0.98 (0.80–1.19)	1.10 (0.92–1.32)	1.16 (0.97–1.39)	0.051
Seaweed	82.0	1 (ref.)	0.97 (0.89–1.04)	0.95 (0.88–1.03)	0.99 (0.92–1.07)	0.717
Potatoes	87.9	1 (ref.)	0.97 (0.91–1.03)	0.99 (0.94–1.05)	0.95 (0.90–1.02)	0.251
Fruit	56.1	1 (ref.)	1.07 (0.91–1.26)	1.11 (0.95–1.29)	1.30 (1.12–1.50)^**^	<0.001
Fats/oils	66.2	1 (ref.)	1.01 (0.89–1.15)	1.05 (0.93–1.18)	1.10 (0.98–1.24)	0.102

CI, confidence interval; PR, prevalence ratio; Q, quartile.

^a^Adjusted for sex, age, body mass index, disease history, medication use, depressive symptoms, perceived chewing ability, smoking, physical activity levels, living alone, food service use, years of education, employment status, driving license. Prevalence ratio calculated by Poisson regression. ^*^P < 0.05, ^**^P < 0.01.

There was a significant trend between low DVS and distance to nearest food store in males (*P* for trend = 0.043) but not in females (*P* for trend = 0.345) (detailed data not shown). There were no significant associations between DVS and distance to nearest food store for each driving status (having a license, *P* for trend = 0.101: not having a license, *P* for trend = 0.193; detailed data not shown).

## DISCUSSION

This cross-sectional study examined the relationship between food environment and diet variety among elderly Japanese living in rural areas. Results showed a significant association between distance from home to nearest food store and lower diet variety.

Distances to nearest food store of 1.8 km or more were associated with low DVS with a significant trend (ie, greater distance related to a higher PR of low DVS, which was confirmed by sensitivity analyses), suggesting that there is a dose-response relationship between greater distance to a food store and low DVS. Our findings that neighborhood food environment significantly related to diet variety is inconsistent with previous studies.^12^^,^^32^ Fukuda et al found no significant relationship between distance to nearest supermarket and DVS among elderly Japanese living alone in suburban and rural areas.^12^ Hamamatsu et al conducted a cross-sectional study among Japanese elderly living in both urban and rural areas and found no association between protein intake and distance to nearest food store.^32^ Our study differed from these previous studies in the content of dietary intake and types of stores that comprised the food environment. Importantly, different types of regions were investigated simultaneously in previous studies, and although targeting both rural and urban areas could increase the chance of generalizability, results would not reflect the uniqueness of any particular region. A previous study demonstrated different effects between urban and rural food environments,^33^ which could be explained by means of transportation for food shopping. In general, there is very little public transport in rural regions in Japan, where most residents use a car to go shopping.^34^ The region covered in our survey was a typical mountainous area of Japan that did not include urban or suburban areas. Our findings reflected a typical food environment in rural Japan, where it is possible that physical distance affects diet variety in the elderly. One particular study, conducted only in an urban area, reported that the distance to supermarkets had a significant positive correlation with DVS score.^15^ This result is consistent with our findings. By not mixing different regions, the effects of a region’s unique physical environmental factors can be properly assessed. Among all food store types evaluated in our study, supermarkets were most strongly associated with diet variety. Furthermore, a previous study demonstrated that the types of food items sold in stores also need to be considered as environmental factors.^35^ It is possible that distance to nearest food stores that sell a variety of food items, such as supermarkets, is important to maintain a higher DVS.

One important finding from our study was that participants who lived more than 1.8 km from the nearest food store had a higher PR for low frequency of both meat and fruit intake. Similarly, Nakamura et al demonstrated an association between absence of a food store within 1 km of home and low frequency of meat and fish intake.^36^ Another systematic review suggested that the relationship between fruit and vegetable intake and distance to food store was due to the fact that fruits and vegetables have short shelf lives or are difficult to store at home.^37^ In our study, most participants (71.0%) got vegetables from their own garden (Table 1). This suggests that for rural elderly, the relationship might be more attributed to in-house vegetable gardens than to distance to nearest food store, a finding we consider could be unique to food environments of rural regions. Therefore, when interventions are conducted to improve the health of rural elderly Japanese, it could be more important to consider meat intake than vegetable intake.

In our study, low DVS was significantly associated with distance to nearest bus stop or railway station in the crude model, but there was no significant association for distance to bus stop in the adjusted model. Previous studies showed a significant relationship between location of bus stop and physical activity habits,^38^^,^^39^ but location of public transportation has never been studied in relation to dietary habits. Although it could be assumed that public transportation is used for food shopping, it is important to examine the relationship between actual transportation means for food shopping and diet variety among the elderly.

There was no significant relationship between DVS and perceived access to food in our study. In previous studies in Japan, perceived access to food was significantly related to low protein intake^32^ and food diversity.^40^ Subjective difficulties to food access are possibly determined by a variety of other factors. The concept of an ecological model, whereby eating behavior is influenced by multiple layers of environmental factors surrounding an individual, suggests that social, physical, and macro-level environmental factors are interconnected.^41^ The content of each factor may vary depending on area of residence, and factors characteristic of rural areas may have been relevant in our study. Most participants in our study had a driving license and it was assumed that many used their own car to go shopping. While subjective difficulty in obtaining food is not related to DVS, people living in rural areas may be unconsciously influenced by distance to food store without feeling inconvenienced by shopping. There is little research on diet variety and subjective food access difficulties in people living in rural areas; therefore, further research is needed to improve dietary habits in the elderly.

Generalizability of our findings should be cautiously undertaken based on several study limitations. First, because this was a cross-sectional study, causal relationships could not be determined. A long-term cohort study on neighborhood food environment and diet variety is required for future research. Second, this study on food environments was limited to rural mountainous areas, and findings cannot be generalized to urban and suburban areas; however, since mountainous areas cover about 70% of Japan’s land area, generalizability can be considered for many areas of Japan. There are not enough studies on neighborhood food environment and dietary variety^12^^,^^15^^,^^40^: similar studies are needed for urban and suburban areas. Third, we did not examine types of food sold at each food store. Although a method was developed to measure the sale of healthy foods in stores,^42^ it did not target the elderly health problem. A future study is needed to measure types of food sold at each store. Fourth, this study was unable to obtain detailed information on participant’s income. It is suggested that income effects diet variety; therefore, our results require careful interpretation as we were unable to adjust for the income effect. Fifth, we did not examine environmental factors other than the physical environment. Schwartz et al reported that social, physical, and macro-level environments relate to each other and affect eating behavior.^41^ A unique social environment may exist in rural areas, which could compensate for the physical environment. In order to learn more about external factors that influence eating behavior, future studies should incorporate the social environment (such as social support or social norms) and the macro-level environment (such as policy actions or cultural norms). Sixth, because there are very few studies in this field, it is difficult to discuss the mechanism behind distance to food store associating with DVS; further studies are required, including conducting qualitative surveys for proof of mechanism. Finally, our research outcome was dietary habits, but not disease or other health outcomes; future studies considering health outcomes caused by DVS decrease are needed.

## CONCLUSION

In this study of a rural Japanese elderly population, greater distance to the nearest food store was significantly related to low DVS and infrequency of meat and fruit intake. Supermarkets and convenience stores, in particular, showed significant associations. Specific interventions may be needed for areas at high risk of low diet variety, such as locations that are far from food stores, especially supermarkets and convenience stores.

## APPENDIX A. SUPPLEMENTARY DATA

eTable 1. Prevalence ratio (95% Confidence Interval) for the low diet variety score (DVS) for the covariates
