# Supplementary material for: Relationship Between Neighborhood Food Environment and Diet Variety in Japanese Rural Community-dwelling Elderly: A Cross-sectional Study
Source: J Epidemiol. 2022 Jun 5;32(6):290–7. doi: 10.2188/jea.JE20200415 (PMC9086309; doi:10.2188/jea.JE20200415)
Supplement: Supplementary file 1 [file je-32-290-s001.pdf]

eTable 1. Prevalence ratio (95% Confidence Interval) for the low diet variety score (DVS) for the covariates

n=1103	No. adults with DVS ≤2 (%)	Crude<br>PR (95% CI)[b]	Adjusted model[a]<br>PR (95% CI)[b]
Sex			
Men	69.4	1 (ref.)	1 (ref.)
female	50.2	0.72 (0.65–0.80)**	0.70 (0.63–0.79)**
Age			
60s	63.7	1 (ref.)	1 (ref.)
70s or older	56.4	0.89 (0.80–0.98)**	0.89 (0.80–0.98)*
Body mass index, kg/m^2			
≤21.4	57.8	1 (ref.)	1 (ref.)
21.5–24.9	59.6	1.03 (0.92–1.15)	1.02 (0.92–1.14)
≥25.0	62.0	1.07 (0.94–1.23)	1.05 (0.92–1.20)
Disease history, yes			
Not having	59.6	1 (ref.)	1 (ref.)
Having one or more	59.3	1.00 (0.89–1.12)	0.97 (0.85–1.10)
Medication use			
None	59.3	1 (ref.)	1 (ref.)
0–4	60.5	1.02 (0.90–1.15)	1.05 (0.91–1.21)
5 or over	56.1	0.95 (0.81–1.11)	0.95 (0.79–1.14)
Depressive symptoms, yes			
No	58.8	1 (ref.)	1 (ref.)
Yes	62.6	1.07 (0.94–1.21)	1.02 (0.90–1.16)
Perceived chewing ability, low			
Moderate	55.4	1 (ref.)	1 (ref.)
Low	66.2	1.19 (1.08–1.31)**	1.15 (1.05–1.27)**
Smoking, yes			
No smoking	58.3	1 (ref.)	1 (ref.)
Smoking	70.4	1.21 (1.05–1.39)**	1.02 (0.88–1.18)
Physical activity levels			
Low	60.9	1 (ref.)	1 (ref.)
Modelate	57.5	0.94 (0.83–1.07)	0.91 (0.80–1.03)
High	47.1	0.77 (0.60–1.00)	0.67 (0.51–0.88)**
Living alone			
No	58.9	1 (ref.)	1 (ref.)
Yes	65.8	1.12 (0.95–1.32)	1.11 (0.94–1.29)
Food service use, yes			
No using	59.7	1 (ref.)	1 (ref.)
Using	53.3	0.89 (0.70–1.14)	0.87 (0.69–1.09)
Years of education			
More than 12 years	51.5	1 (ref.)	1 (ref.)
12 years or less	61.1	1.18 (1.02–1.37)**	1.17 (1.01–1.36)*
Employment status			
No	57.3	1 (ref.)	1 (ref.)
Yes	61.9	1.08 (0.98–1.19)	1.02 (0.92–1.13)
Driving license			
Having	59.9	1 (ref.)	1 (ref.)
Not having	55.3	0.92 (0.79–1.09)	1.12 (0.94–1.33)

CI, confidence interval; DVS, Diet variety score; PR, prevalence ratio.

[a]Adjusted for distance to the nearest food store by quartiles, sex, age, body mass index, disease history, medication use, depressive symptoms, perceived chewing ability, smoking, physical activity levels, living alone, food service use, years of education, employment status, driving license.

[b]Prevalence ratio calculated by Poisson regression. $^{*}P < 0.05$, $^{**}P < 0.01$.
